# Gender inequity in postpartum hemorrhage: A public health issue

**DOI:** 10.1002/ijgo.70526

**Published:** 2025-09-24

**Authors:** Anne‐Beatrice Kihara, Monica Oguttu, Ahmet Metin Gülmezoglu, Albaro Jose Nieto‐Calvache, Akaninyene Eseme Ubom, Cherrie Evans, Diana Ramasauskaite, Zechariah J. Malel, Dietmar Schlembach, Ines Nunes, Bo Jacobsson, Jolly Beyeza‐Kashesya, Ferdousi Begum, Alison Wright, for the FIGO Committee on Childbirth and Postpartum Hemorrhage

**Affiliations:** ^1^ Department of Obstetrics and Gynaecology, Faculty of Medicine University of Nairobi Nairobi Kenya; ^2^ Kisumu Medical and Education Trust Kisumu Kenya; ^3^ Concept Foundation Geneva Switzerland; ^4^ Departamento de Ginecología y Obstetricia Fundación Valle del Lili Cali Colombia; ^5^ Facultad de Ciencias de la Salud Universidad ICESI Cali Colombia; ^6^ Department of Obstetrics, Gynaecology and Perinatology Obafemi Awolowo University Teaching Hospitals Complex Ile‐Ife Nigeria; ^7^ Helping Mothers Survive, Technical Leadership and Innovations, Jhpiego Baltimore Maryland USA; ^8^ Center of Obstetrics and Gynecology Medical Faculty of Vilnius University Vilnius Lithuania; ^9^ School of Medicine University of Juba Juba South Sudan; ^10^ Clinicum Vivantes Neukölln Berlin Germany; ^11^ Department of Obstetrics and Gynecology Gaia/Espinho Local Health Unit Porto Portugal; ^12^ CINTESIS—Center for Health Technology and Services Research University of Porto Porto Portugal; ^13^ Department of Medical Sciences University of Aveiro Aveiro Portugal; ^14^ Department of Obstetrics and Gynecology Sahlgrenska Academy, University of Gothenburg Gothenburg Sweden; ^15^ Department of Obstetrics and Gynecology, Western Health Care Region Sahlgrenska University Hospital Gothenburg Sweden; ^16^ Department of Genetics and Bioinformatics, Division of Health Data and Digitalisation Institute of Public Health Oslo Norway; ^17^ Mulago Specialised Women and Neonatal Hospital Kampala Uganda; ^18^ Department of Obstetrics & Gynecology Institute of Woman and Child Health Dhaka Bangladesh; ^19^ Royal Free London NHS Foundation Trust London UK; ^20^ University College Hospital, University College London London UK

**Keywords:** access to care, gender, inequalities, inequity, maternal mortality, postpartum hemorrhage

## Abstract

Postpartum hemorrhage (PPH), a leading cause of maternal mortality globally, disproportionately affects women in low‐ and middle‐income countries (LMICs), highlighting the deep‐rooted gender related inequities in healthcare access, quality, and outcomes. Despite being largely preventable and treatable, PPH continues to claim the lives of thousands of women annually, because of systemic failures, including inadequate maternal health infrastructure, under‐resourced healthcare systems, and sociocultural norms that devalue women's health. Gender inequity is manifested in delayed care‐seeking, a lack of decision‐making autonomy, limited access to skilled birth attendants, and emergency obstetric care. Moreover, implicit biases and structural discrimination often limit investment in women‐centered health interventions. This issue is compounded by socioeconomic disparities, educational gaps, and the underrepresentation of women's health priorities in policy and research agendas. Addressing PPH through a gender‐equity lens is imperative to improve maternal health outcomes and achieve global health equity. This paper underscores the urgent need for integrated, gender‐sensitive public health strategies to mitigate the burden of PPH and protect the rights and lives of women.

## 1 BACKGROUND

Too many women have had their lives cut short due to avoidable deaths during pregnancy and childbirth.^1^ Although the number of maternal deaths has declined globally, sub‐Saharan Africa (SSA) has shown minimal progress in reducing maternal deaths.^2^ Globally, postpartum hemorrhage (PPH) accounts for over 27% of all maternal deaths, and approximately 38% among women in low‐ and middle‐income countries (LMICs).^3^ Despite being generally preventable and treatable, an estimated 70 000 women continue to die annually from PPH, with 99% of these deaths occurring in LMICs, and an estimated two‐thirds in SSA.^4^ Substantial progress has been made toward improving maternal and child health outcomes, including the adoption of evidence‐based policies and legal frameworks, promoting male involvement in maternal healthcare, strengthening the role and capacity of midwives and the wider workforce, improving education and access to family planning, and adopting community‐based interventions to improve the demand for healthcare services.

However, all these efforts have not yet yielded the desired results, highlighting the need to refocus on PPH as a gender issue.^5^ Several studies have identified gender as a fundamental driver of maternal and child health,^5^, ^6^ with many citing a potential relationship between gender inequality (as captured by the Gender Inequality Index [GII]), maternal mortality, and health expenditure per capita.^7^ Gender inequity and discrimination, which often manifest in inequities in household decision‐making, labor, caregiving, and social norms dictating the status of women,^5^ impede women's access to health care, thereby contributing to poor health outcomes.^8^ An analysis of trends in maternal mortality and morbidity indicates a higher number of maternal deaths in countries where women are least likely to have skilled attendance at birth.^9^ Similarly, within these countries, the poorest and least educated women are the most vulnerable to maternal death and disability.^10^ Therefore, a woman's chances of dying or becoming disabled during pregnancy and childbirth are closely connected to her socioeconomic status, cultural norms and values, and the geographical remoteness of her home.^11^

This paper explores gender biases and their relationship with maternal mortality, particularly in SSA. While many investments have been made to strengthen health systems for women, available evidence points to the need to complement these health investments with broader interventions aimed at shifting social ‘norms’ and addressing gender inequalities at the macro, societal, and household levels.^7^ The contextual gender‐related issues contributing to excess maternal mortality from PPH in Africa can be applied to the ‘three‐delays’ model through a gender‐equity lens.^9^

## 2 DELAY 1: DECIDING TO SEEK CARE

Despite well‐defined societal gender roles, men and women often have different expectations that affect how they negotiate healthcare decisions and utilize health services at the household level. Societal ‘norms’, cultures, and traditions reinforce gendered roles, lowering women's status and limiting their autonomy, which negatively affects maternal healthcare utilization.^12^, ^13^

Sociocultural factors, such as traditional beliefs, cultural practices, and gender ‘norms’ related to childbirth and menstruation, can impact how PPH is perceived, prevented, and managed within communities.^14^ Men who make these healthcare decisions are typically unaware of holistic maternal health information; thus, their decisions are not always in the best interest of women or their infants.^14^ Similarly, studies indicate that low literacy levels and a lack of equitable job opportunities among women in LMICs have resulted in a high dependency on men in healthcare‐seeking decisions.^15^

Because male partners and family members can play a supportive role in PPH prevention and management, it is necessary to engage men in broader maternal health education, encouraging their involvement in antenatal care and childbirth for better maternal outcomes.^14^ This is especially true in largely patriarchal societies, where women need to seek the consent of their male partners to access healthcare services and interventions.

## 3 DELAY 2: REACHING AN APPROPRIATE FACILITY

Women need appropriate access to antenatal care during pregnancy, skilled care during childbirth, and support during the weeks following childbirth.^15^ However, many women are unable to access high quality maternal healthcare because of low socioeconomic status and the inability to meet childbirth‐related costs. Financial autonomy and access to resources can be crucial for enabling women to seek and obtain the care they need.^16^

Timely access is also hindered by difficult terrain and long distances to travel to healthcare facilities. These challenges are harder to navigate when the woman lacks control over her financial or transport resources. Consequently, births often occur at home or on the way to the healthcare facility, because the decision to seek care is made when it is already too late.

## 4 DELAY 3: RECEIVING ADEQUATE CARE

Access to skilled birth attendants is not always feasible in low‐resource settings. Additionally, essential medicines and supplies for life‐saving interventions may be unavailable. Disempowered women face potential delays when seeking care, and ineffective referral systems can result in late hospital arrivals. Women with life‐threatening complications such as PPH may not survive if they encounter an unresponsive health system. Even after overcoming the first two delays, women may face financial barriers, including the need to purchase essential medical supplies that are unavailable at the hospital.

Furthermore, while long waiting times at health facilities present a key barrier to receiving adequate care,^15^ gender biases among healthcare providers (HCPs) can influence the quality of care provided to women experiencing PPH. Training healthcare providers in gender‐sensitive communication, respectful maternity care, and empathy can improve patient‐provider interactions and outcomes.^17^ For example, understanding the relationship between the gender of the HCP providing clinical care to the patient and the patient's experience with the services is crucial for designing and delivering gender‐responsive care. Training local midwives, ensuring that health centers are suitably equipped to provide safe and family‐friendly births, and improving referral systems between health centers and hospitals are key strategies for improving maternal health care, including PPH management. PPH is a time‐sensitive condition that requires timely access to skilled providers, appropriate equipment, and compassionate, motivated care.

To effectively address gender inequity, the following approaches are recommended (Box 1).

BOX 1 **Recommended approaches for addressing gender inequity in PPH care.**

***Empowerment and decision‐making:*** Women's empowerment and decision‐making autonomy are crucial for positive maternal health outcomes, including PPH prevention and management. Women must be empowered to make decisions about their health and childbirth, especially regarding the identification of danger signs, seeking timely care, communicating their needs and preferences, and exercising their autonomy.^5^, ^9^

***Healthcare provider bias and communication:*** Women constitute over 70% of the healthcare workforce at all levels of the health system. However, globally, they hold less than 30% of leadership and governance positions.^18^, ^19^ Addressing this gender disparity is crucial, as empowering women to occupy managerial or senior executive positions can significantly improve health system outcomes. Women in these roles have qualifications, experience, and perspective based on their lived experiences related to pregnancy and childbirth. The inclusion of gender‐sensitive communication, respectful maternity care, and empathy in healthcare worker training, both in‐ and pre‐service, is key to improving patient‐provider interactions and outcomes.^17^, ^20^

***Sociocultural factors:*** Sociocultural factors such as traditional beliefs, cultural practices, and gender norms related to childbirth and menstruation impact how PPH is perceived, managed, and prevented within communities.^14^ Understanding and addressing these factors are essential for effective interventions.

***Role of male partners and family support:*** Male partners and family members can play supportive roles in PPH prevention and management.^14^ Men should be engaged from the outset, starting with family planning, to promote respectful partnerships and support women's choices regarding the timing of birth. Their involvement should continue through antenatal care, birth, and the postpartum period to improve outcomes.

***Data collection and research:*** Gender‐disaggregated data collection and analysis are critical for understanding the gender‐specific factors influencing PPH incidence, outcomes, and access to care. Research incorporating gender perspectives can inform targeted interventions and policy recommendations. Research should incorporate both quantitative and qualitative methods, such as participatory action research (PAR), to generate evidence for local decision‐making. A significant problem in many low‐resource settings is the reliance on manual hospital records and filing systems, which results in missing case records and patient data. Therefore, more attention should be paid to gender mainstreaming in women's health research. While women are affected disproportionately, differently, and uniquely, reports on clinical trials, interrogation of their life experiences, and employment of technologies and innovations are limited. Additionally, increasing funding opportunities and successful grant awards for female educators and researchers are essential.^21^

***Policy and programmatic interventions:*** Integrating gender‐equity and equality in maternal health policies, guidelines, and programs is essential for addressing the underlying determinants of PPH and promoting gender‐equity healthcare delivery.^9^ This includes nutrition to prevent anemia among pregnant women and ensuring equitable access to life‐saving interventions, such as uterotonics, blood transfusions, and surgical interventions.

Healthcare policies should include the promotion of emergency obstetric care services, development of primary and secondary levels of health care, adequate maternity staffing, training and retraining of maternity staff. They should also prioritize good road and transportation networks to curb the three phases of delay that prevent women from accessing effective and timely PPH preventive and treatment care.

## 5 CONCLUSION

Increasing evidence now points to a relationship between gender inequity, women's empowerment, health expenditure, and maternal mortality. In particular, countries with higher expenditure on health tend to have a lower GII and maternal death rate.

While direct factors such as distance to health centers, lack of health insurance, and unaffordable fees, are significant contributors to maternal deaths, broader societal interventions are also crucial. Increasing financial resources for education, reproductive health, sanitation, and transportation, are likely to improve gender equity and reduce maternal deaths. Empowering women through education, providing them with resources, and ensuring their autonomy in decision‐making are essential for improving access to life‐saving interventions in PPH management. Therefore, these measures are crucial in reducing maternal mortality and morbidity.

Eradicating gender inequity in PPH is not only a moral imperative, but a public health necessity. Every woman, regardless of her location or social status, deserves a safe birth and a healthy future. Let us commit to a world where no woman dies from preventable causes in childbirth.

## AUTHOR CONTRIBUTIONS

ABK, MO, FB, and JBK contributed to the conceptualization of the paper. ABK, AMG, AJNC, AEU, CE, DR, ZJM, DS, IN, BJ, and AW contributed to the content, writing, editing, and finalization of the manuscript. The second round of review and revision was coordinated by AW and implemented by MO to produce the final version of the manuscript. All authors read and approved the final manuscript.

## FUNDING INFORMATION

No funding was received for this study.

## CONFLICT OF INTEREST STATEMENT

The authors have no conflicts of interest.

## Data Availability

Data sharing is not applicable to this article as no new data were created or analyzed in this study.
